# Maternal Snuff Use and Smoking and the Risk of Oral Cleft Malformations - A Population-Based Cohort Study

**DOI:** 10.1371/journal.pone.0084715

**Published:** 2014-01-15

**Authors:** Anna Gunnerbeck, Anna-Karin Edstedt Bonamy, Anna-Karin Wikström, Fredrik Granath, Ronny Wickström, Sven Cnattingius

**Affiliations:** 1 Department of Women's and Children's Health at Karolinska Institutet, Neonatal Research Unit, Karolinska University Hospital Solna, Stockholm, Sweden; 2 Department of Medicine, Clinical Epidemiology Unit at Karolinska Institutet, Stockholm, Sweden; 3 Department of Women's and Children's Health at Uppsala University, Uppsala, Sweden; The Ohio State University, United States of America

## Abstract

**Objective:**

To determine if maternal use of snuff (containing high levels of nicotine, low levels of nitrosamines and no combustion products) is associated with an increased risk of oral cleft malformations in the infant and whether cessation of snuff use or smoking before the antenatal booking influences the risk.

**Method:**

A population-based cohort study was conducted on all live born infants, recorded in the Swedish Medical Birth Register from 1999 through 2009 (n = 1 086 213). Risks of oral clefts were evaluated by multivariate logistic regression analyses (using adjusted odds ratios, with 95% confidence intervals [CI]).

**Results:**

Among 975 866 infants that had information on maternal tobacco use, 1761 cases of oral clefts were diagnosed. More than 50% of the mothers who used snuff or smoked three months prior pregnancy stopped using before the antenatal booking. Almost 8% of the mothers were smoking at the antenatal booking and 1,1% of the mothers used snuff. Compared with infants of non-tobacco users, the adjusted odds ratios (95% CI) of any oral cleft for infants of mothers who continued to use snuff or to smoke were 1.48 [1.00–2.21] and 1.19 [1.01–1.41], respectively. In contrast, in infants of mothers who stopped using snuff or stopped smoking before the antenatal booking, the corresponding risks were not increased (adjusted odds ratios [95% CI] were 0.71 [0.44–1.14] and 0.88 [0.73–1.05], respectively).

**Conclusion:**

Maternal snuff use or smoking in early pregnancy is associated with an increased risk of oral clefts. Infants of mothers who stopped using snuff or stopped smoking before the antenatal booking had no increased risk of oral cleft malformations. Oral snuff or other sources of nicotine should not be recommended as an alternative for smoke-cessation during pregnancy.

## Introduction

Oral cleft malformations are among the most common congenital malformations worldwide, with a birth prevalence of approximately 1.7/1000 live born babies, with ethnic and geographic variations [1]. Oral clefts result from disrupted fusion of the facial prominences during the first trimester. The etiology seems to be multifactorial, and previous studies have indicated maternal smoking as an important risk factor [1]–[5]. We lack knowledge of whether the effect of smoking is generated by nicotine per se or by combustion products in tobacco smoke. Despite lack of knowledge of possible adverse effects of nicotine on fetal development, nicotine-replacement therapy, NRT, is sometimes recommended as a means of smoking cessation also for pregnant women [6], [7]. Studies on NRT during pregnancy are few and because of lack of compliance, adverse effects are difficult to study [6].

By studying the use of Swedish snuff (containing mainly nicotine) and smoking during pregnancy concurrently, we can differentiate between the effects caused by nicotine and those linked to combustion products in tobacco smoke. Swedish oral snuff, or “snus”, contains similar or higher levels of nicotine and lower levels of tobacco specific nitrosamines than cigarettes and American snuff, and its use as a harm reduction drug for smoking cessation is internationally debated [8]–[10] [11]–[13]. Habitual smokers and snuff users have similar maximal nicotine levels in plasma, but in snuff users the peak of nicotine concentration is longer than it is in smokers (approximately 1 hour compared to 30 minutes). The accumulated concentration of nicotine may, therefore, be higher in snuff users than in smokers [14], [15]. Recent studies on maternal snuff use during pregnancy have demonstrated adverse effects, such as increased risks of preterm birth, stillbirth and neonatal apnea [16]–[18].

Should nicotine be the causative agent underlying the increased risk of oral clefts seen in infants of smoking mothers, the risk would also be increased in infants of snuff using mothers. In addition, women who stop smoking or stop using snuff before pregnancy or in very early pregnancy should reduce their risk of having infants with oral clefts. To our knowledge, previous studies have neither investigated the association between prenatal exposure to smokeless tobacco and the risk of oral cleft malformations, nor studied whether change of exposure to tobacco influences this risk.

In Sweden, data on snuff use and smoking before pregnancy and in early pregnancy are collected in the nationwide Swedish Medical Birth Register. This enabled us to study associations between maternal snuff use and smoking habits and risks of oral cleft malformations, and whether cessation of snuff use or cigarette smoking reduces these risks.

## Methods

### Ethical approval

The population-based register study was approved by the Research Ethics Committee of Karolinska Institutet, Stockholm (no. 2012/366-32). This is a population-based register study, and consent was not obtained by individual participants. It was specifically waived by the approving IBR because of the very large study population.

### Study population

The nationwide Swedish Medical Birth Register contains data on 98% of all births in Sweden, including demographic data, information on reproductive history, pregnancy, delivery, and the neonatal period [19]. Our study population included 1 086 213 live born infants recorded in the Medical Birth Register from 1999 through 2009. By means of the unique personal identification number, the Medical Birth Register can be linked with other data sources. The Patient Register includes nationwide information on diseases and procedures on all in-patient care from 1987, and information on hospitals' outpatient care is provided since 2001 [20]. Since 1997, diagnoses in both the Patient Register and the Birth Register are classified according to the International Classification of Diseases, 10^th^ Revision (ICD-10). The Education Register includes annually updated information about the highest level of formal education for all Swedish residents. The Register of Total Population includes information about country of birth.

### Exposures

Information about tobacco use is collected in the MBR at the antenatal booking, which generally occurs at 8 to 12 gestational weeks and before 15 weeks of gestation in 95% of all pregnancies [21]. Information on both snuff use and cigarette smoking can be obtained from 1999 in MBR. Women are asked about present tobacco habits and about tobacco habits three months before pregnancy. Information about tobacco use is categorized as non-use, snuff use (yes or no), moderate smoking (1 to 9 cigarettes per day), and heavy smoking (≥10 cigarettes per day).

To investigate the potential effect of change in tobacco exposure, we combined information about tobacco use before pregnancy and in early pregnancy to categorize women as: 1) non-tobacco users (nonusers of snuff and cigarettes before and in early pregnancy); 2) women who stopped using snuff (used snuff three months before pregnancy but not at the antenatal booking); 3) current snuff users (used snuff before and in early pregnancy); 4) women who stopped smoking (smoked three months before pregnancy but not at the antenatal booking); and 5) current smokers (smoked both before and in early pregnancy). In these analyses we excluded infants of mothers who changed substance of tobacco use or started to use tobacco (n = 2 996), who were dual users before and/or in early pregnancy (n = 3 112), as well as infants of mothers with missing information on tobacco use before and/or in early pregnancy (n = 110 347). The analyses of tobacco exposures and change in tobacco exposures and risks of oral cleft malformations included 89% (n = 969 758) of the study population. Information on tobacco use three months before pregnancy and in early pregnancy is presented in Table 1.

**Table 1:** Tobacco habits before pregnancy and in early pregnancy in mothers of 1 086 213 live-born infants born in Sweden 1999 to 2009.

		BEFORE PREGNANCY					
		Total No. (%)	Nonuser (n)	Snuff user (n)	Smoker (n)	Snuff user and smoker (n)	Missing (n)
**EARLY PREGNANCY**	**Total No. (%)**	1 086 213 (100)	773 625 (71.2)	21 994 (2.0)	185 248 (17.2)	2 895 (0.2)	102 451 (9.4)
	**Nonuser (n)**	917 900 (84.5)	765 145	12 834	97 776	1 476	40 669
	**Snuff user (n)**	11 461 (1.0)	1 237	8 859	732	597	36
	**Smoker (n)**	92 092 (8.5)	953	74	85 144	295	5 626
	**Snuff user and smoker (n)**	746 (0.1)	11	16	207	510	2
	**Missing (n)**	64 014 (5.9)	6 279	211	1 389	17	56 118

From the Medical Birth Register, we retrieved information on maternal age at delivery, parity, whether the mother was co-habiting with the father-to-be, maternal diseases, sex of the infant, and multiple births. We included information about maternal pre-gestational diabetes (ICD-10 codes E10-14, O241-O243), chronic hypertension (ICD-10 codes O10-11 and I10-15), and preeclampsia (ICD-10 codes O14-15). From the Education Register, we collected information on mother's number of years of formal education completed as of January 1st, 2010. From the Register of Total Population, we retrieved information about mother's country of birth, which was categorized into Nordic (Sweden, Finland, Norway, Denmark, and Iceland) or non-Nordic. Other variables were categorized according to Table 2 and Table 3.

**Table 2:** Maternal characteristics and tobacco use in early pregnancy.

	Nonuser (n = 765 145) Rate (%)	Stopped using snuff (n = 12 834) Rate (%)	Current snuff use (n = 8 859) Rate (%)	Stopped smoking (n = 97 776) Rate (%)	Current smoker (n = 85 144) Rate (%)	Changed tobacco use (n = 2 996) Rate (%)	Snuff user and Smoker (n = 3 112) Rate (%)	Missing of tobacco use (n = 110 347) Rate (%)
**Maternal age (yrs)**								
≤19	1.2	1.4	1.7	4.3	5.6	3.2	5.0	2.4
20–24	10.2	15.2	13.9	23.0	23.7	15.3	25.4	12.3
25–29	30.3	33.2	28.5	34.6	28.7	31.1	29.1	30.5
30–34	37.4	33.8	33.4	26.1	24.4	30.5	24.9	35.2
≥35	21.0	16.3	22.6	12.0	17.6	19.8	15.7	19.7
**Parity**								
1	41.9	59.0	40.0	59.9	41.2	44.8	61.8	44.2
2–3	52.3	38.3	52.2	36.4	47.1	48.1	33.0	49.7
4	3.8	2.1	5.4	2.7	7.1	4.8	3.2	4.0
≥5	2.1	0.7	2.5	1.0	4.6	2.3	2.0	2.1
**Co- habitant with the father**								
No	3.9	5.2	6.8	9.1	16.3	8.6	12.7	5.4
Yes	96.1	94.9	93.2	90.9	83.8	91.4	87.3	94.6
Missing	1.2	0.9	1.3	1.2	1.8	1.2	0.8	43.0
**Mothers country of birth**								
Non Nordic	18.3	4.0	4.0	17.2	15.2	13.1	4.2	16.1
Nordic	81.7	96.0	96.0	82.8	84.8	86.9	95.8	83.9
Missing	1.2	0.1	0.3	0.9	1.2	0.9	0.2	2.4
**Education**								
≤11 years	20.5	17.0	32.0	33.4	58.1	34.8	37.5	28.3
12 years	25.5	31.0	31.6	35.9	28.3	29.1	33.9	23.4
≥13 years	54.0	52.0	36.4	30.7	13.7	36.1	28.6	48.3
Missing	2.9	0.6	1.0	2.3	2.9	2.2	0.7	4.4

**Table 3:** Maternal characteristics and rates of oral cleft malformations in the newborn.

	All births	Oral clefts	
	(n = 1 086 213)	(n = 1 985)	
	Total No.	(n)	Rate/l 000
**Tobacco use in early pregnancy**			
Non-user	917 900	1 616	1.8
Snuff user	11 461	31	2.7
Cigarette smoker	92 092	207	2.2
Dual user	744	2	2.7
Missing	64 014	129	2.0
**Tobacco use 3 months before and in early pregnancy**			
Nonuser	765 145	1 357	1.8
Stopped using snuff	12 834	17	1.3
Current snuff user	8 859	25	2.8
Stopped smoking	97 776	165	1.7
Current smoker	85 144	197	2.3
Other change of tobacco exposure^a^	2 996	6	2.0
Dual user^b^	3 112	4	2.0
Missing^c^	110 347	214	1.9
**Maternal age (years)**			
≤19	21 120	29	1.4
20–24	138 443	258	1.9
25–29	332 151	609	1.8
30–34	379 966	653	1.7
≥35	214 205	436	2.0
Missing	328	0	0
**Parity**			
1	447 480	842	1.8
2–3	542 416	1007	1.8
4	43 057	87	2.0
≥5	23 260	49	2.1
**Family situation**			
Co-habitant with father	967 403	1 748	1.8
Not co-habitant with father	57 480	113	2.0
Missing	59 582	124	2.0
**Mothers country of birth**			
Non Nordic	186 657	253	1.4
Nordic	886 042	1720	1.9
Missing	13 514	12	0.1
**Education**			
≤11 years	269 183	534	2.0
12 years	280 235	541	1.9
≥13 years	504 469	870	1.7
Missing	32 326	40	1.2
**Chronic hypertension**			
Yes	6 888	16	2.3
No	1 079 325	1 969	1.8
**Preeclampsia**			
Yes	32 597	78	2.4
No	1 053 616	1 907	1.8
**Prepregnancy diabetes**			
Yes	4 747	6	1.3
No	1 081 466	1 979	1.8
**Sex**			
Male	558 599	1 135	2.0
Female	527 292	850	1.6
Missing	322	0	0
**Birth**			
Singleton	1 054 185	1 907	1.8
Multiple	32 015	78	2.4
Missing	13	0	0

^a^ Changed substance of tobacco or started to use snuff or to smoke.

^b^ Dual users before and/or in early pregnancy.

^c^ Information on tobacco habits was missing before and/or in early pregnancy.

### Outcomes

Information was retrieved from the MBR on the initial assessment of the infant's health that was made immediately after birth by the midwife or doctor, and the final assessment that was made before discharge from hospital. Diagnoses were noted by the doctor at discharge. By linkage to the Patient Register, we could also collect data from infants who were diagnosed with oral clefts later during their first year. We included information about any oral cleft malformations (that is cleft lip and/or cleft palate) using ICD-10 codes Q35-Q37. Because of suggested differences in genetic origin [1], [5], [22], we also analysed oral clefts subdivided into cleft lip with or without cleft palate (ICD-10 codes Q36 and Q37) and isolated cleft palate (Q35). Oral clefts are associated with other congenital defects, and oral clefts were therefore stratified into oral clefts combined with or without other malformations. We identified 1985 infants with an oral cleft malformation. Of these, 81% (n = 1612) were recorded in the Medical Birth Register and 19% (n = 373) were only registered in the Patient Register (of whom 97 were obtained from outpatient visits).

### Statistical analysis

The risk of oral clefts in the newborn in relation to maternal tobacco exposure was assessed by studying infants of mothers who used snuff or smoked, in comparison with infants of non-tobacco users. We also compared the risk of oral clefts in infants of mothers who had stopped using snuff or stopped smoking before the antenatal booking with infants of non-tobacco users. Comparisons were performed by unconditional logistic regression and results were presented as crude and adjusted odds ratios (ORs), using 95% confidence intervals (CI). ORs were adjusted for variables that in previous studies were associated with the exposure and/or the outcome [23], [24]. Odds ratios were estimated crude and with adjustments for maternal and birth characteristics, including maternal age, parity, family situation (co-habiting with father-to-be or not), mother's country of birth, maternal education, maternal diseases (hypertension, preeclampsia and diabetes), sex of the newborn, and single or multiple birth. These variables were categorized as shown in Table 2 and Table 3. In addition, to account for the variation in frequency of recorded diagnoses and tobacco use, we also adjusted for calendar year of infant's birth, which was categorized into 1999–2000, 2001–2003 and 2004–2009.

In order to adjust for the dependence introduced by the fact that mothers may contribute with more than one child, the Generalized Estimation Equation method was applied [25]. All analyses were performed using procedure PROC GENMOD in SAS version 9.2 (SAS Institute, Inc., Cary, NC).

## Results

Compared to nonusers, tobacco users (both snuff users and smokers) were to a greater extent teenage mother, less educated, multiparous and not living with the father-to-be. These patterns were more pronounced in smokers than in snuff users. During the study period, about 8% of the mothers were smokers at the antenatal booking and 1,1% of the mothers used snuff. More than 50% of women who were snuff users or smokers three months before pregnancy had stopped using tobacco at the time of the antenatal booking. Approximately, 40% of previous snuff users and 46% of previous smokers were current users in early pregnancy. Less than 2% of snuff users and smokers changed form of tobacco use, had missing information on tobacco use or were dual users (Table 1). Compared to non-tobacco users, mothers who stopped using tobacco were to a greater extent teenage mothers, not living with the father-to-be, less educated and primiparas (Table 2).

Infants of current snuff users and smokers had increased rates of oral clefts in comparison to non-tobacco users (Table 3). In contrast, infants of mothers who had stopped using snuff or stopped smoking before antenatal booking had almost the same, or even lower, rates of oral clefts as infants of non-tobacco users. High maternal age (≥35 years), a Nordic country of birth, chronic hypertension, preeclampsia, male sex, and multiple births were associated with increased rates of oral clefts, while pre-pregnancy diabetes was associated with a reduced rate.

In the crude analysis, infants of mothers who used snuff or smoked in early pregnancy were at higher risk of any oral cleft malformation than infants of non-tobacco users (Table 4). In the adjusted analysis, these risks were slightly attenuated, and the risk related to snuff use was of borderline significance. Compared with infants of non-tobacco users, the risk of any oral cleft malformation was not increased among infants of mothers who stopped using snuff or stopped smoking before registration to antenatal care.

**Table 4:** Maternal tobacco use and risks of oral cleft malformations in the newborn.

Tobacco Use	No.	Rate	Odds Ratio (95% Confidence Interval)	
		(%)	Crude	Adjusted^a^
Nonuser	1 357	0.18	1.00	1.00
Current snuff user	25	0.28	1.59 (1.07–2.37)	1.48 (1.00–2.21)
Stopped using snuff	17	0.13	0.75 (0.46–1.21)	0.71 (0.44–1.14)
Current smoker	197	0.23	1.31 (1.12–1.52)	1.19 (1.01–1.41)
Stopped smoking	165	0.17	0.95 (0.81–1.12)	0.88 (0.73–1.05)
Nonuser	860	0.11	1.00	1.00
Current snuff user	17	0.19	1.71 (1.06–2.76)	1.61 (1.00–2.61)
Stopped using snuff	12	0.09	0.83 (0.47–1.47)	0.77 (0.44–1.37)
Current smoker	132	0.16	1.38 (1.15–1.66)	1.33 (1.09–1.63)
Stopped smoking	101	0.10	0.92 (0.75–1.13)	0.84 (0.67–1.05)
Nonuser	497	0.06	1.00	1.00
Current snuff user	8	0.09	1.39 (0.69–2.80)	1.26 (0.63–2.55)
Stopped using snuff	5	0.04	0.60 (0.25–1.45)	0.59 (0.24–1.43)
Current smoker	65	0.08	1.18 (0.91–1.52)	0.98 (0.73–1.32)
Stopped smoking	64	0.07	1.01 (0.78–1.31)	0.94 (0.71–1.24)

^a^ Adjusted model : maternal age, parity, education, living with father-to-be or not, hypertension, diabetes, preeclampsia, sex of newborn, birth (singleton or multiple), for variation of diagnosis frequency and mothers' country of birth.

We also analysed the different oral cleft malformations separately, but the numbers of infants with specific malformations born to snuff users were small, which affected the precision of the estimates (Table 4). Compared with non-tobacco users, infants of snuff users and smokers had increased risk of being born with cleft lip with or without cleft palate. Neither infants of snuff users, nor infants of smokers were at increased risk of isolated cleft palate malformation. Compared with infants of non-tobacco users, infants of mothers who stopped using snuff or stopped smoking before antenatal booking were not at increased risk of any specific oral cleft malformation.

In infants with oral clefts, other malformations occurred in 35% (n = 11) of infants of snuff users in early pregnancy, in 25% (n = 51) of infants of smokers and in 28% (n = 446) of infants of non-tobacco users. Chi^2^-test did not show any significant difference between these groups (p = 0.4).

## Discussion

Our population-based cohort study indicated that maternal snuff use in early pregnancy was associated with increased risks of oral cleft malformations in the infant. In consistency with previous studies [3], maternal smoking was also associated with increased risk of oral clefts. Infants of mothers who stopped using snuff or stopped smoking before the antenatal booking were not at increased risk of oral clefts. These findings support the hypothesis that early fetal nicotine exposure may be teratogenic in humans.

Animal studies of prenatal nicotine exposure have shown disturbances in the development of the fetal nervous system and the cardiorespiratory control [26], [27]. In vitro studies by Zhao et al. showed that nicotine affects the embryogenesis in a dose-dependent manner by increasing Ca^2+^ -levels and activating the reactive oxidative stress system, causing congenital malformations by inducing apoptosis and excessive cell death [28]. Nicotine has been reported to inhibit palate fusion in vitro in a dose-dependent manner [29], and to induce teratogenic effects of palate formation in mice [30], [31]. There is also some evidence of interaction between genotype and maternal smoking with respect to risk of oral cleft malformations [32]–[34].

A limitation of the study is the borderline significance in the adjusted analyses of current snuff users and smokers and risk of malformations. Oral cleft malformation, being one of the most common malformations, is still a rare condition. About 1.1% of Swedish women use snuff and less than 6% smoke in early pregnancy [35]. The number of cases of oral cleft malformations is a limitation to the study. However, our results, showing an association between prenatal smoking and an overall risk of oral clefts, are in agreement with most studies [4], [5], [36], [37]. Previous literature is not consistent as to whether there is an association with maternal smoking for the subgroups cleft lip (with or without cleft palate) and isolated cleft palate [2], [8], [33], [37]–[41]. In contrast to two previous studies using data from the Swedish Medical Birth Register (1983–97 and 1983–92, respectively) [5], [42], we could not show an association between maternal smoking or snuff use and isolated cleft palate. The number of cases of isolated cleft palate in the study was small and lack of association may have been due to lack of power. In Sweden, there has been a dramatic decline in maternal smoking during pregnancy: from approximately 32% in 1983 to 8% in 2009 [43]. One speculative explanation for these discrepant results may be that the dose of nicotine required to cause damage is higher for isolated cleft palate than for other cleft malformations.

Oral clefts result from failure of migration or fusion of the facial prominences between the fourth and eighth weeks after conception. During this period, the embryo is most susceptible to environmental factors predisposing to oral clefts. The earlier the interruption or interference takes place, the greater the defect [1]. If tobacco exposure interferes with this process, then women who stop using snuff or stop smoking before pregnancy or in very early pregnancy should reduce their risks of having offspring with oral clefts. We lack information of when, in the time span three months before pregnancy to antenatal booking (generally at eight to twelve gestational weeks), women stopped using snuff or stopped smoking. Given that more than 50% of women who used snuff or smoked three months before pregnancy stopped using tobacco before antenatal booking, we find it likely that most of these women did so because they were planning a pregnancy or when learning they were pregnant.

The common substance in cigarette smoke and snuff is nicotine, but they also contain tobacco specific nitrosamines, TSNA. The production process of Swedish “snus” differs from that of American oral moist snuff, making the levels of tobacco specific nitrosamines considerably lower in “snus” (mean TSNA 1,1 ug/g tobacco) [8] than in cigarettes (mean TSNA 2,54 ug/g tobacco) [9] and American snuff (TSNA levels between 4,87–90,02 ug/g) [10]. Findings from epidemiological studies have shown an association between prenatal exposure to nitrate from drinking water and exposure to nitrosable drugs during pregnancy and neural tube defects in the infant [44]–[46]. According to Huber et al., there is insufficient knowledge to suggest an association between dietary nitrosamines and congenital malformations [47]. A possible interaction between prenatal exposure to nitrosable drugs, dietary nitrite and tobacco specific nitrosamines has, to our knowledge, not been studied.

Higher risks of oral clefts were found among infants prenatally exposed to snuff than among infants prenatally exposed to smoking. Maximal nicotine plasma levels are similar in smokers and snuff users and slightly lower in subjects using nicotine-replacement therapy. Whereas smoking generates a short peak of high plasma nicotine levels, corresponding peaks generated by snuff and nicotine-replacement therapy have longer durations [15]. If nicotine is teratogenic in humans, long-term exposure to high nicotine levels may be more harmful than short-term exposure. Today, there is increasing knowledge of harmful effects of snuff use during pregnancy [16]–[18], while large-scale safety studies on adverse effects of nicotine replacement therapy during pregnancy are lacking [6]. A Danish National Birth Register study showed no increased overall risk of congenital malformations in connection with maternal smoking but showed an increased risk of congenital malformations, especially musculoskeletal, with nicotine substitutes such as gum, patches and inhalers. The increased risk of maternal smoking and oral clefts was in accordance with previous studies, while the effect of snuff use was not studied [48].

The nationwide Swedish Medical Birth Register provides access to information about snuff use and smoking both three months before pregnancy and in early pregnancy. This enabled us not only to differentiate between the effects of nicotine and those of tobacco combustion products, but also to investigate if change of exposure influenced risks. We are unaware of any study that has investigated associations between prenatal exposure to any kind of smokeless tobacco and risks of oral clefts as well as between change of smoking habits and risks of oral clefts. Data about tobacco exposures three months before pregnancy and in early pregnancy were collected at the antenatal booking before the first ultrasonic scan, which precludes recall bias.

We only have self-reported data on tobacco habits. Smokers were recorded as moderate and heavy smokers (1–9 and ≥10 cigarettes per day, respectively) in the registry, but we have no knowledge about level of consumption or pattern of snuff use. We lack information of the exact time at which mothers using tobacco three months prior pregnancy but not at the antenatal booking actually stopped doing so. Because of the extensive knowledge of the negative effects of smoking during pregnancy, there is also a risk of underreporting of tobacco use. George et al. showed that prospectively collected self-reported information on smoking habits in early and late pregnancy was valid in Sweden. However, the misclassification rate was highest among those who reported cessation of smoking during pregnancy and most recently had stopped smoking. [49]. Self-reported information on snuff use during pregnancy has not been validated. If snuff users or smokers were recorded as non-tobacco users, we may have underestimated the tobacco-related risks of oral cleft malformations. Information on tobacco habits of the father-to-be or second hand smoking was not possible to obtain.

The slightly lower overall risk in women who stop using snuff compared with non-tobacco users is not significant and may be due to chance. However, women who stop using tobacco may also be more health-conscious in other respects. They have higher education than those mothers who continued to use tobacco. We lack information of possible protective factors, such as dietary folic acid intake [50], and possible risk factors for oral clefts, including alcohol use, maternal use of anti-epileptics and other drugs [1]. Although high alcohol consumption is associated with increased risk of oral clefts [51], [52], adjusting for maternal alcohol use did not influence the smoking-related risk of oral clefts in previous studies [2], [8], [40].

The limited number of oral clefts in infants of snuff users also hampered our possibilities to investigate the association between snuff use and risks of oral cleft subtypes, including syndromic and non-syndromic oral clefts.

## Conclusion

Our study shows that both maternal snuff use and smoking during pregnancy are associated with increased risk of oral cleft malformations in the newborn. Both nicotine and nitrosamines per se may have teratogenic effects. Consequently, snuff and other sources of nicotine are not to be regarded as safe alternatives to smoking during pregnancy. Our study shows the importance of abstaining from any form of nicotine during pregnancy to reduce the risk of oral cleft malformation.
